# Impact of Oral Administration of *Lactiplantibacillus plantarum* Strain CNCM I−4459 on Obesity Induced by High-Fat Diet in Mice

**DOI:** 10.3390/bioengineering10101151

**Published:** 2023-10-01

**Authors:** Elsa Jacouton, Stanislas Mondot, Philippe Langella, Luis G. Bermúdez-Humarán

**Affiliations:** Institut National de Recherche pour l’Agriculture et l’Environnement (INRAE), AgroParisTech, Micalis Institute, Université Paris-Saclay, 78350 Jouy-en-Josas, France; elsa.jacouton@inrae.fr (E.J.); stanislas.mondot@inrae.fr (S.M.); philippe.langella@inrae.fr (P.L.)

**Keywords:** *Lactiplantibacillus plantarum*, glucose sensitivity, lipid metabolism, microbiota, high-fat diet

## Abstract

Recent evidence suggests that some lactobacilli strains, particularly *Lactiplantibacillus plantarum*, have a beneficial effect on obesity-associated syndromes. Several studies have investigated probiotic challenges in models of high-fat diet (HFD)-induced obesity, specifically with respect to its impact on hepatic and/or adipocyte metabolism, gut inflammation and epithelial barrier integrity, and microbiota composition. However, only a few studies have combined these aspects to generate a global understanding of how probiotics exert their protective effects. Here, we used the probiotic strain *L. plantarum* CNCM I−4459 and explored its impact on a mouse model of HFD-induced obesity. Briefly, mice were administered 1 × 10^9^ CFUs/day and fed HFD for 12 weeks. Treatment with this strain improved insulin sensitivity by lowering serum levels of fasting glucose and fructosamine. Administration of the probiotic also affected the transport and metabolism of glucose, resulting in the downregulation of the hepatic *Glut-4* and *G6pase* genes. Additionally, *L. plantarum* CNCM I−4459 promoted a decreased concentration of LDL-c and modulated hepatic lipid metabolism (downregulation of *Fasn, Plin,* and *Cpt1α* genes). Probiotic treatment also restored HFD-disrupted intestinal microbial composition by increasing microbial diversity and lowering the ratio of Firmicutes to Bacteroidetes. In conclusion, this probiotic strain represents a potential approach for at least partial restoration of the glucose sensitivity and lipid disruption that is associated with obesity.

## 1. Introduction

Non-communicable diseases (NCDs) are the leading cause of mortality worldwide and are favored by a combination of genetics and lifestyle factors. One such NCD is obesity, which correlates with several metabolic syndromes such as insulin resistance, type-2 diabetes, and certain cancers (such as colorectal cancer). Obesity and overweight correspond to an excess of fat accumulation, mainly caused by a prolonged imbalance between energy intake and energy expenditure (World Health Organization). Several factors, such as genetic and environmental ones, influence obesity, as well as a Western diet (rich in simple sugars and fat and poor in fiber), which has been recognized as an “obesogenic” diet. In addition, obesity and metabolic syndrome are characterized by altered gut microbiota, inflammation, and barrier dysfunction [1,2,3,4]. Obesity affects around 13% of the adult population worldwide and has a high cost of treatment, making it an important public health issue (World Health Organization). At the level of individuals, nutritional interventions are typically the preferred strategy for preventing obesity. Several studies have reported the impact of diet on gut microbiota dysbiosis [2,5] and the means by which this can affect obesity. Our increased understanding of the numerous ways in which the Western diet, human biology, and gut microbiota interact has opened new possibilities for the use of probiotics as a therapeutic strategy.

Probiotics are live microorganisms that, when administered in adequate amounts, confer a health benefit on the host (Food and Agriculture Organization of the United Nations [6]). The most studied probiotic strains belong to the group of lactic acid bacteria (LAB). Several health benefits have been attributed to LAB (mainly lactobacilli strains), including the regulation of the host immune response and the epithelial barrier homeostasis, as well as the modulation of the gut microbiota and metabolic functions [7,8,9,10,11]. In the last two decades, different strains of *Lactobacillus* spp. have been extensively studied as an alternative treatment for intestinal inflammation (e.g., chemically induced colitis and colorectal cancer) [12,13,14], intestinal hyper-permeability [15], or even metabolic disorders induced by HFD [16,17]. In addition, several studies have reported specific beneficial health effects of different strains of *Lactobacillus* spp. on the control of body weight, glucose tolerance, and hyperlipidemia [18,19]. In particular, strains of *Lactiplantibacillus plantarum* have been shown to help reduce obesity and ameliorate metabolic syndromes in mouse models of HFD-induced obesity, thus representing good candidates for obesity prevention strategies [16,20,21]. In addition, a controlled, randomized, double-blind trial demonstrated that *L. plantarum* strains had a beneficial effect in lowering cholesterol levels [22]. These studies have highlighted the pivotal role that the liver and adipocytes play in lipid metabolism to prevent HFD-induced obesity. However, although microbiota dysbiosis is generally altered in metabolic diseases (10.1136/gutjnl-2020-323071, 10.1186/s13073-016-0303-2), only a few addressed how *L. plantarum* strains impact microbiota in HFD mice model [23,24] and the mechanisms underlying the beneficial effects mediated by *L. plantarum* are then still poorly understood.

*L. plantarum* strain CNCM I−4459 is known to maintain epithelial intestinal integrity in a mouse model of colitis (IL10-deficient) [25]. In addition, based on a preliminary study, the strain has been shown to activate in vitro (i.e., a cellular model using Hutu 80 cells) the expression of *Pyy*, a gene with an effect on satiety and metabolism (Supplementary Figure S1). We thus decided to explore the impact of strain CNCM I−4459 in a model of HFD-induced obesity in mice.

## 2. Materials and Methods

### 2.1. Bacterial Strain and Growth Conditions

*Lactiplantibacillus plantarum* CGMCC No.1258 (*L. plantarum* CNCM I−4459) (CGMCC No.1258, Novanat, Shanghai, China) was isolated from the feces of a healthy child and kindly provided by Indigo Therapeutics. It was grown in MRS (Man Rogosa and Sharpe, Difco, Le-Pont-de-Claix, France) medium at 37 °C overnight in aerobic conditions. To prepare the live bacterial inoculum, bacteria were washed two times with PBS and spun down at 3000 g, and the pellet was suspended in PBS to a final concentration of 5 × 10^9^ colony-forming units (CFUs)/mL in PBS with 15% glycerol.

### 2.2. Animal and Experimental Design

Male C57BL/6J mice (6–8 weeks old; Janvier SAS, St Berthevin, France) were maintained at INRAE animal facilities (4 mice per cage). Mice were assigned to three groups: two groups of mice (*n =* 16) were fed a high-fat diet (HFD; 60 Kj % fat, Ssniff, Soest, Germany), and one group (*n =* 8) received a control diet (CD) (13 Kj % fat, Ssniff) for twelve weeks (diet composition is described in Supplementary Figure S2). The assay was performed in two independent experiments in the same conditions. Weight and food intake (g/cage) were measured once a week throughout the experiment. The food efficiency ratio (FER) was calculated as follows: body weight gain/calorie intake. Once a day, mice were orally administered either 1 × 10^9^ CFUs of *L. plantarum* or PBS. Mice were euthanized by cervical dislocation on day 84. Colon, ileum, liver, adipose tissues, and sera were collected and stored under conditions appropriate for further analyses. Feces were collected in the morning, frozen in nitrogen immediately after collection, and stored at −80 °C before processing. All animal experiments were approved by the local INRAE ethics committee and the French Ministry of Research (approval 2015070115416973).

### 2.3. Oral Glucose Tolerance Test (OGTT)

Mice were fasted for 6 h (by removal of food and bedding) before OGTT analysis. Glucose solution (2 g/Kg) was orally administered. Blood glucose levels were measured at time 0 (before glucose gavage) and 15, 30, 60, and 120 min after glucose gavage using a One Touch glucometer (Roche, Meylan, France). The area under the curve (AUC) was calculated following the trapezoidal rule. Insulin levels were detected using a Mouse Ultrasensitive Insulin Elisa (Alpco, Salem, NH, USA) at T0 and T30 min. The homeostatic model assessment of insulin resistance (HOMA IR) was calculated according to the formula: (fasting glucose (T0) [mg/dL] × fasting insulin (T0) [ng/mL])/405.

### 2.4. Measurement of Plasma Parameters

Blood samples were collected in tubes and centrifuged (for 10 min at 3000 rpm), and plasma samples were stored at −80 °C. Measurements of plasma free fatty acid (FFA) (NEFA C, Wako Chemicals GmbH, Neuss, Germany), low-density lipoprotein cholesterol (LDL-c) (ABX Pentra LDL direct CP, Horiba, Montpellier, France), triglyceride (TG) (ABX Pentra Triglyceride CP, Horiba, Montpellier), high-density lipoprotein cholesterol (HDL-c) (ABX Pentra HDL direct CP, Horiba, Montpellier), and protein glycation (ABX Pentra Fructosamine CP, Horiba, Montpellier) were performed by Anexplo (Toulouse, France) using a Pentra 400 Analyzer.

### 2.5. Gene and Protein Expression Analysis

Ileum, colon, liver, visceral, and epididymal adipose tissue (vAT, eAT) samples were stored at −80 °C in RNAlater (Sigma-Aldrich, Saint-Louis, MO, USA). RNA was extracted from tissues using RNeasy Mini Kit (Qiagen, Hilden, Germany) according to the manufacturer’s instructions. A Trizol treatment was added for lipid-rich tissues. cDNA synthesis was performed from 1 μg of RNA using the High-Capacity cDNA Reverse Transcription kit (Applied Biosystems, Foster, CA, USA) according to the manufacturer’s instructions. RT-qPCR was carried out with Taqman probes (β-*Actin*; Mm01963702_S1, *Lep*; Mm00434759_m1, *Ap-2/Fabp-4*; Mm00445878_m1, *Insig-2*; Mm00460121_m1, *Ppar-α*; Mm00440939_m1, *G6pase*; Mm00839363_m1, *Glut-4*; Mm00436615_m1, *Adrp/Plin*; Mm00475794_m1, *Zo-1*; Mm00493699_m1, *Tnf-α*; Mm00443258_m1, *Fasn*; Mm00662319_m1, *Cpt1a*; Mm01231183_m1, *Glut-2;* Mm00446229_m1, *Cd36*; Mm00432303_m1, *Gcgc-1*; Mm00553234_m1, *Insig-2*; Mm00460121_m1, *Nrf-2*; Mm00477784_m1, *Demoglein-2*; Mm00809994_s1, *Claudin-2*; Mm00516703_s1, *Claudin-5*; Mm00727012_s1) (Life Technologies, Villebon-sur-Yvette, France) according to the manufacturer’s instructions in a reaction volume of 20 μL using an ABI Prism 7700 (Applied Biosystems, USA) thermal cycler. ADD: cycling conditions and mix composition. To quantify and normalize the expression data, we used the 2ΔΔCt method with ß-Actin as the endogenous reference gene.

Total proteins were extracted from ileum sections using T-Per buffer (Thermoscientific, Rockford, IL, USA) and protease inhibitor mixture (Roche, Pensberg, Germany), with mechanical lysis from a Precellys homogenizer (Ozyme, Saint-Cyr-l’Ecole, France) (2 runs of 4500 g for 30 s). Supernatants were collected and subjected to ELISA (Mabtech, Nacka Strand, Sweden) for the quantification of IL-17, IL-22, and TNF-α according to the manufacturer’s instructions. For Western Blot, 10 µg of protein were loaded on precast Mini Protean TGX Stain Free gels (4–20%, Biorad, Marnes-la-Coquette, France). Blot (Trans Blot Turbo Transfer system, Biorad) was incubated with β-Actin rabbit (2 µg/mL, Cell Signaling, Danvers, MA, USA) and Zo-1 rabbit (1/250e, Invitrogen, Calsbarg, CA, USA) antibodies. A secondary goat anti-rabbit antibody was used (1/5000e, Jackson ImmunoResearch, Cambridge, UK). Blot was revealed with Clarity Western ECL substrate kit (Biorad, Marnes-la-Coquette, France) using Chemidoc Imager (BioRad, Marnes-la-Coquette, France).

### 2.6. Microbial DNA Extraction and Amplification

DNA was extracted from stool as previously described in Lamas et al. [26]. The resulting DNA pellet was washed with 70% ethanol, dried, and resuspended in 50 μL of Tris–EDTA (TE) buffer. DNA suspensions were stored at −20 °C until amplification.

The 16S rRNA gene (V3 region) was PCR-amplified using 1 U of Taq, 1× buffer, 1.5 mM MgCl_2_, 0.4 mg/mL BSA, 0.2 mM dNTPs, and 5 pmol of 341F (CCTACGGGAGGCAGCAG) and 806R (GGACTACHVGGGTWTCTAAT) primers. Amplicons were further modified for MiSeq sequencing as described in Bartram et al. [27]. PCR products were loaded on a 1.5% agarose gel, and positive PCR reactions were normalized using the SequalPrep normalization kit (ThermoFisher).

A 16S rDNA amplicon library was sequenced in the Surette lab and the Farncombe Metagenomics Facility on a MiSeq machine using the 2 × 250 bp V3 kit. Any remaining adapter/primer sequences were trimmed, and reads were checked for quality (≥30) and length (≥200 bp) using cutadapt [28]. Reads were further corrected for known sequencing errors using SPAdes [29] and then merged using PEAR [30]. A total of 3,259,918 reads was produced, with an average of 83,588 ± 15,247 reads per sample. Sequencing data are deposited in NCBI under the accession number PRJNA663256.

OTUs were identified using a Vsearch pipeline [31] designed to dereplicate (–derep_prefix–minuquesize 2) and cluster (–unoise3) the merged reads, as well as check for chimeras (uchime3_denovo). Taxonomic classification of OTUs was performed using the classifier from the RDPTools suite [32]. Representative OTU sequences were taxonomically assigned using the RDP classifier with a SAB score ≥ 0.5.

### 2.7. Microbiota Composition Analysis

Statistical analyses were conducted using the R programming language and software (R Development Core Team 2012), specifically using the packages gplots, gdata, vegan [33], ade4 [34], phyloseq [35], and phangorn [36]. OTU counts were normalized via simple division to their sample size and then multiplication by the size of the smallest sample. α-diversity and richness were estimated using the OTU table data and the functions “diversity” and “estimateR”. A distance matrix for β-diversity analysis was computed using the “vegdist” function and the Bray–Curtis method. Principle coordinate analysis was conducted on the distance matrix data using “dudi.pco”. Differential enrichment in bacterial taxa among groups was assessed using the linear discriminant analysis (LDA) effect size (LEfSe) algorithm [37]. Kruskal–Wallis rank sum tests and post hoc pairwise Wilcoxon rank sum tests were used to detect differences between groups of variables. *p* values were corrected as necessary using the false discovery rate correction.

### 2.8. Measurement of Short-Chain Fatty Acids (SCFAs)

Concentrations of fecal SCFAs (acetate, butyrate, propionate, isobutyrate, and valerate) were measured with a mass spectrometer (Université de Nantes, IRS-UN, France) using gas chromatography coupled with spectrometry.

### 2.9. Statistical Analysis

Statistics were calculated with Prism software (version 9.4). A normality test (Shapiro–Wilk test) was systematically performed on the data. In the case of normal distribution, one-way ANOVA, followed by Tukey’s multiple comparison test, was used. In case of lack of normal distribution, data were analyzed using the Kruskal–Wallis test, followed by Dunn’s multiple comparison test. The level chosen for statistical significance was 5%.

## 3. Results

### 3.1. L. plantarum CNCM I−4459 Enhanced oral Glucose Tolerance by Inhibiting Glucose Metabolism

**Figure 1:**
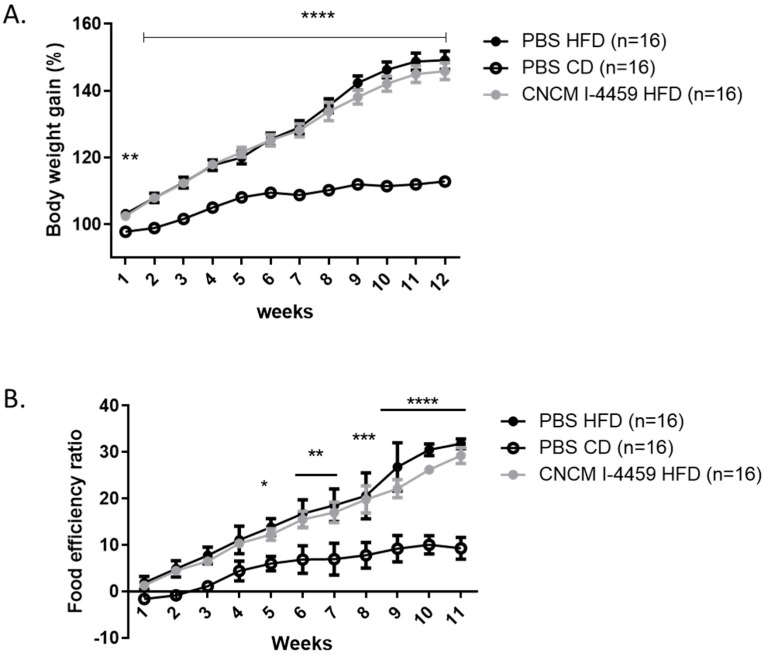
Effect of *L. plantarum* CNCM I−4459 supplementation on body weight (**A**); food efficiency ratio (**B**)**.** Mice were separated into 3 groups: mice treated with PBS and fed with CD (control diet) (PBS-CD group, *n* = 16), mice treated with PBS and fed with HFD (high-fat diet) (PBS-HFD group, *n* = 16), and mice treated with *L. plantarum* CNCM I−4459 (1 × 10^9^ CFU/day) and fed with HFD (*L. plantarum* CNCM I−4459-HFD group, *n* = 16). *, **, ***, **** represent a *p* < 0.05, *p* < 0.01, *p* < 0.001, *p* < 0.0001 (Two ways ANOVA, Bonferroni post-test) compared to CD-fed mice. Data are represented as mean ± SEM.

**Figure 2:**
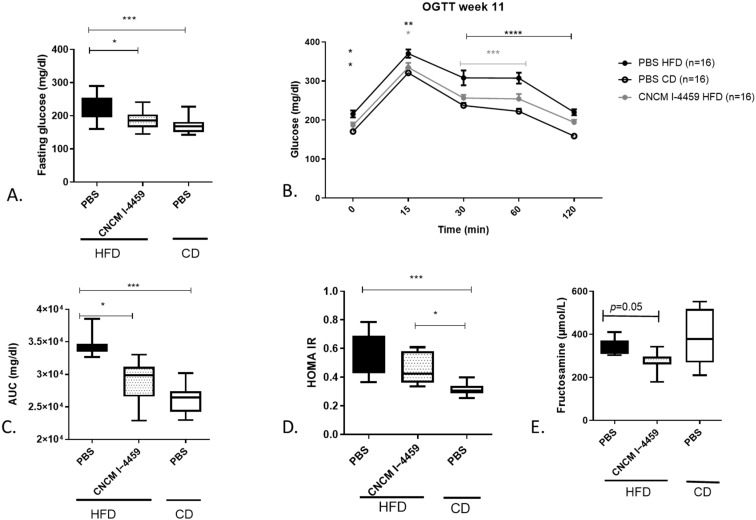
**Effect of *L. plantarum* CNCM I−4459 supplementation on oral glucose tolerance test:** (**A**) fasting glucose level; (**B**) serum glucose measurement after glucose administration (Two-way ANOVA, Bonferroni post-test) compared to CD-fed mice (grey color indicates a comparison to HFD-fed mice), (**C**) AUC; (**D**) the index of homeostasis model assessment for insulin resistance (HOMA IR = (Fasting glucose (T0) [mg/dL] × fasting insulin (T0) [ng/mL])/405); (**E**) serum level of fructosamin. Data are represented as mean ± SEM. PBS-HFD *n* = 16, PBS-CD *n* = 16 and *L. plantarum* CNCM I−4459-HFD group, *n* = 16 except for HOMA IR and fructosamin, *n =* 8 mice per group). Data were analyzed with one-way ANOVA, followed by Tukey’s multiple comparison, except for C, which was analyzed with Kruskal–Wallis test (Dunn’s post hoc test). Data are represented as box and whiskers plots (mean, minimal, and maximum values), and *, **, ***, **** represent a *p* < 0.05, *p* < 0.01, *p* < 0.001, *p* < 0.0001.

Body weight and food intake were monitored weekly. This experiment was performed twice with a total number of 12–16 animals per group. As expected, PBS-HFD mice gained significantly more weight than PBS-CD-treated mice with no effect of *L. plantarum* either on body weight gain or on food efficiency ratio (FER) (measured as the ratio of body weight gain/calorie intake), cumulative food intake, or genes involved in satiety (*Pyy* and *Gcg-1*) (Figure 1, Figure S3). We then performed OGTT on mice that had been fasting for 6 h. HFD-fed mice exhibited a higher fasting glucose level (Figure 2A, *p* ≤ 0.001), as well as higher levels at every time tested during the OGTT (Figure 2B). Treatment with *L. plantarum* for 12 weeks significantly reduced fasting glucose levels and glucose levels from T15 min to T60 min after glucose challenge, to a range that was similar to that of CD-treated mice (Figure 2B, *p* ≤ 0.05 and *p* ≤ 0.001). Consequently, the AUC decreased in mice treated with this strain (Figure 2C, *p* ≤ 0.05) compared to PBS-HFD mice. Additionally, serum insulin levels were measured in fasting mice and 30 min after glucose administration. Only PBS-CD mice exhibited lower insulin levels at T0 and T + 30 min compared to PBS-HFD mice (Figure S3B). The HOMA-IR index revealed no significant insulin sensitivity in *L. plantarum*-treated mice (Figure 2D) compared to PBS-HFD mice. Finally, HFD-fed mice treated with *L. plantarum* CNCM I−4459 exhibited lower plasma fructosamine levels (HbA1c) than control mice (Figure 2E, *p* = 0.05).

**Figure 3:**
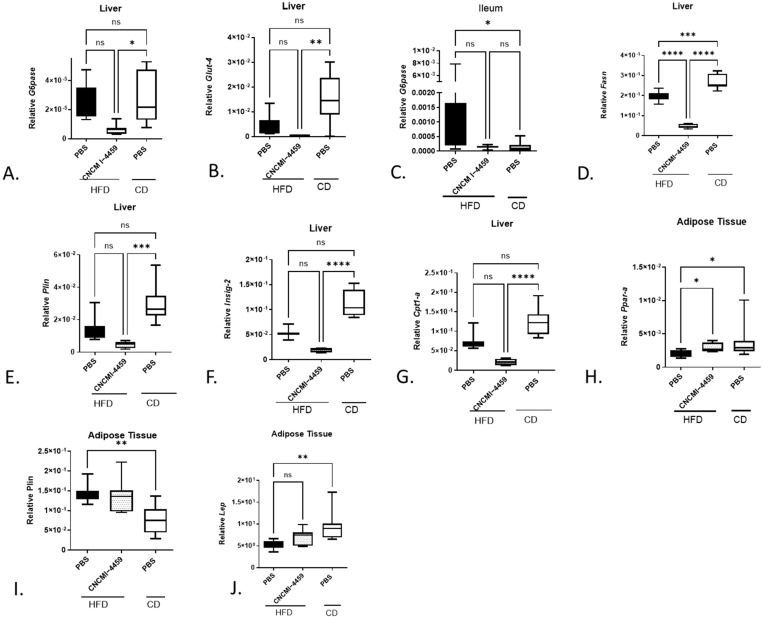
**Effect of *L. plantarum* CNCM I−4459 supplementation on glucose and lipids metabolism:** (**A**) hepatic mRNA *G6pase* expression; (**B**) hepatic mRNA *Glut4* expression; (**C**) ileal mRNA *G6pase* expression; (**D**) hepatic mRNA *Fas* expression; (**E**) hepatic mRNA *Plin* expression; (**F**) hepatic mRNA *Insing-2* expression; (**G**) hepatic mRNA *Cpt1-a* expression; (**H**) adipocyte mRNA *Pparα*; (**I**) adipocyte mRNA *Plin*; (**J**) adipocyte mRNA *Leptin* expression. *Pparα, Leptin,* and *Plin* were measured in eAT. Data are represented as box and whiskers plots (mean, minimal, and maximum values) and compared to PBS-administered HFD-fed mice. *, **, *** and **** represent *p* < 0.05, *p* < 0.01, *p* < 0.001 and *p* < 0.0001, respectively. ns= no significant. Data were analyzed with Kruskal–Wallis test (Dunn’s post hoc test) except for (**A**,**D**,**I**) (one-way ANOVA, followed by Tukey’s multiple comparison) for 8 mice per group.

To decipher the molecular mechanisms underlying the improvement in glucose sensitivity, we measured the expression in the liver of the gluconeogenic *G6pase* gene and the main glucose transporters, *Glut-2* and *Glut-4*. Although no significant modification was observed in PBS-HFD mice, treatment with *L. plantarum* significantly reduced the expression of *G6pase* (*p* ≤ 0.05) and the insulin-dependent *Glut-4* transporter (*p* ≤ 0.01) compared to CD mice (Figure 3A,B). No change was observed in the expression of the bidirectional transporter *Glut-2* (Supplementary Figure S4A). Compared to CD, HFD resulted in significant upregulation of ileal *G6pase*, but this effect was not observed in the ileum of mice treated with this strain (Figure 3C).

Altogether, these results suggested that supplementation with *L. plantarum* CNCM I−4459 partially restored glucose sensitivity in HFD-fed mice, in part by downregulating hepatic and ileal metabolism and insulin-dependent transport.

### 3.2. Treatment with L. plantarum CNCM I−4459 Decreased Concentrations of Circulating Lipids and Modulated Hepatic Lipid Metabolism

Serum levels of TG, FFA, HDL-c, and LDL-c were analyzed in the different groups of mice (Table 1). As expected, HFD-fed mice harbored significantly increased levels of serum HDL-c, LDL-c, and TC compared to the control group, suggesting the onset of dyslipidemia. Interestingly, the HFD group treated with *L. plantarum* CNCM I−4459 had similar levels of LDL-c as PBS-CD mice and significantly lower than PBS-HFD mice (*p* ≤ 0.0001).

Several studies have reported an increase in lipolysis and/or decreased lipogenic activity in obese subjects. For this reason, we also analyzed the hepatic and adipocyte expression of genes involved in lipogenesis and β-oxidation. As described in Figure 3D, the lipogenic gene *Fasn* (*p* ≤ 0.0001) was downregulated in the livers of mice treated with *L. plantarum* compared with PBS-HFD mice. Interestingly, though, expression of the lipolytic gene *Cpt1-a* (*p* ≤ 0.0001) and the lipogenic *Plin* (*p* ≤ 0.001) gene also decreased compared to PBS-CD mice (Figure 3G). Furthermore, the *Insig-2* gene expression (an insulin-dependent inhibitor of the lipogenic *Srebp* genes) was downregulated in *L. plantarum*-HFD mice compared to PBS-CD mice (Figure 3D, (*p* ≤ 0.0001). However, no significant up-regulation of other hepatic lipolytic and lipogenic genes tested in HFD-fed mice was observed (Supplementary Figure S4B–G). Also, no particular change in lipid metabolism in adipose tissues was observed, except for down-regulation of the adipogenic genes *Ppar-α* (in eAT), the lipogenic *Plin* (in eAT), and *leptin* (in vAT) in HFD-fed mice (Figure 3H–J). Interestingly, treatment with *L. plantarum* CNCM I−4459 significantly increased the expression of *Ppar-α* (Figure 3H, *p* ≤ 0.05). In addition, we assessed thermogenesis by measuring *Ucp1-α* expression and found significant regulation in all HFD-fed mice (Supplementary Figure S4H). Altogether, these data suggested that oral administration of *L. plantarum* resulted in a global downregulation of hepatic lipid metabolism (lipolysis and lipogenesis).

### 3.3. Treatment with L. plantarum CNCM I—4459 Reduce ileal Inflammation Affect Moderately Epithelial Junctions

**Figure 4:**
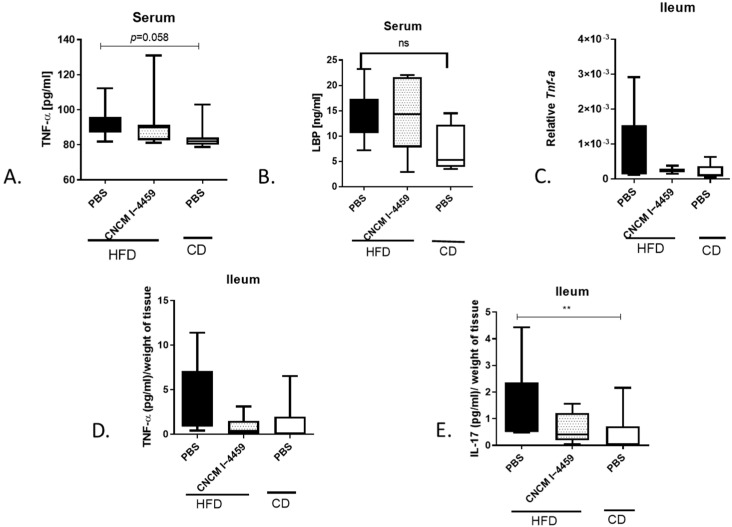
**Assessment of systemic and intestinal inflammation:** (**A**) serum TNF-α level; (**B**) serum LBP level; (**C**) mRNA level of ileal *Tnf*-α; (**D**) ileal protein TNF-α level; (**E**) ileal protein IL-17 level. Data are represented as box and whiskers plots (mean, minimal, and maximum values) for 8 mice per group. Data were analyzed with Kruskal–Wallis test (Dunn’s post hoc test) and compared to PBS-administered HFD-fed mice. LBP; LPS (Lipopolysaccharide)-binding protein. ns = no significant. ** represent a *p* < 0.01.

HFD-induced obesity is often associated with inflammation. Here, systemic inflammation and gut permeability were assessed by measuring serum levels of TNF-α and LBP proteins. PBS-HFD mice had higher levels of TNF-α compared to PBS-CD mice (Figure 4A, *p* = 0.058) and a slight (but not significant) increase in LBP (Figure 4B, ns); no change was observed with *L. plantarum* treatment. In addition, ileal inflammation was assessed by measuring mRNA levels of *Tnf*-α (Figure 4C) as well as protein levels of TNF-α and IL-17. As shown in Figure 4E, PBS-HFD mice exhibited a significant increase in IL-17 protein expression compared to PBS-CD mice (*p* ≤ 0.01). However, no modification was observed either with *L. plantarum* treatment or in other tissues, including adipose tissue (Supplementary Figure S5).

Since inflammation is often linked with disturbances in intestinal permeability, we measured the expression of tight junction (*Claudin-2*, *Claudin-5*, *Occludin*, and *Zo-1*) genes in the ileum and colon. In colon samples, only ZO-1 protein and *Claudin-5* mRNA expression were dysregulated in PBS-HFD mice compared to their littermates (Supplementary Figure S6A–E). *L. plantarum*-fed mice only increased the expression of ZO-1 protein compared to PBS-HFD mice. In the ileum samples, HFD administration appeared to increase mRNA expression of *Zo-1* (*p* ≤ 0.05) and *Claudin-2* (*p* ≤ 0.01) but reduced ileal gene expression of *Occludin* (*p* ≤ 0.05) with no modification with *L. plantarum* administration (Supplementary Figure S6F–I). In parallel, we assessed the levels of cytokine IL-22, a key cytokine in epithelium homeostasis. In the ileum samples, mice fed with HFD exhibited a higher expression of Il-22 expression with no effect of the strain (Supplementary Figure S6J). Regarding *Zo-1*, because of the well-known link between inflammation, epithelium integrity, and oxidative stress, *Nrf-2* gene expression was also measured in ileum samples, but no difference was observed in *L. plantarum*-fed mice (Supplementary Figure S6K).

### 3.4. L. plantarum CNCM I −4459 Treatment Partially Reversed the Effect of Diet on Gut Microbiota Composition

**Figure 5:**
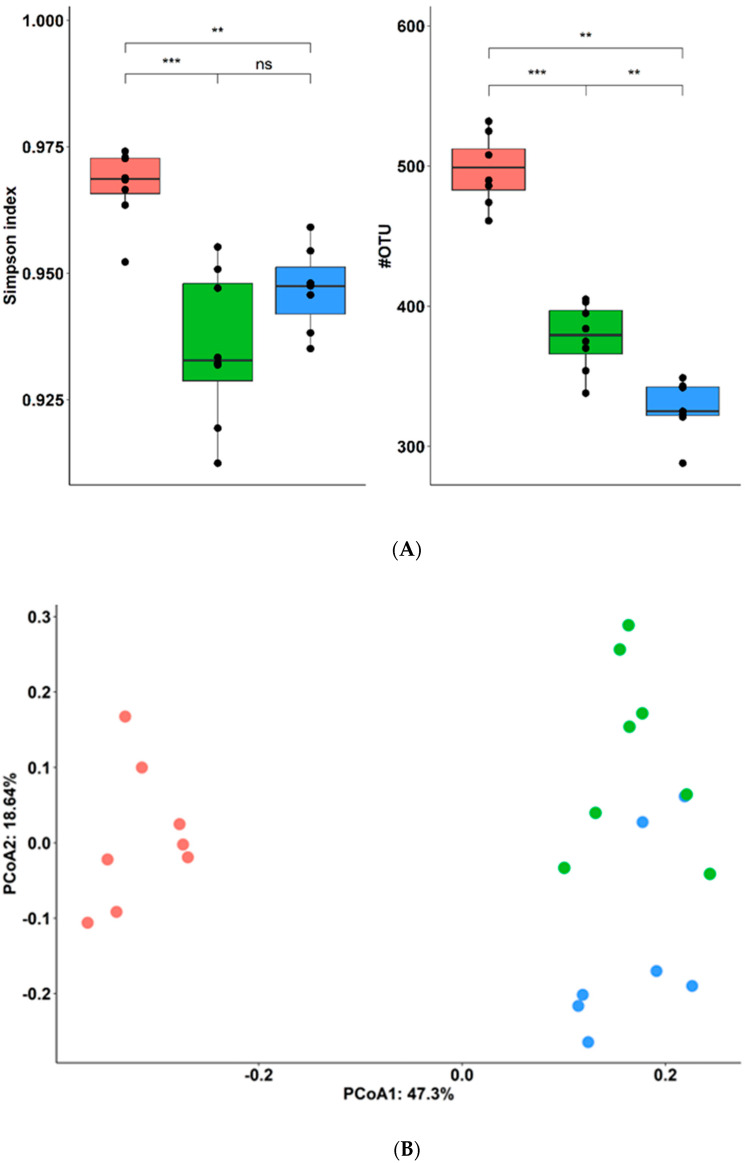

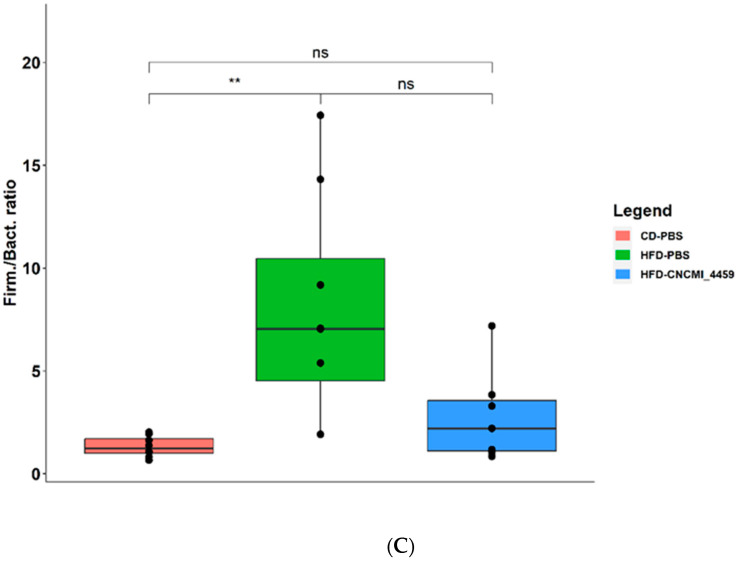
**Effect of *L. plantarum* CNCM I −4459 on the gut microbiota structure:** (**A**) Microbial diversity (** and *** respectively represent *p* < 0.01 and *p* < 0.001, ns = no significant, Kruskal–Wallis rank sum test (pairwise Wilcoxon rank sum post hoc test)); (**B**) principal coordinate analysis (PCoA) plot depicting the inter-individual variability based on the microbiota composition. Each dot is a unique composition of a single fecal sample. Distances between dots highlight the degree of similarity among samples. Difference between groups of samples stratified according to diets and/or CNCM I −4459 was tested by PERMANOVA. Samples in red correspond to mice fed with CD (*n* = 8 mice), samples in blue correspond to mice fed with HFD and treated with *L. plantarum* CNCM I −4459 (*n* = 7 mice), and samples in green correspond to mice fed with HFD (*n* = 8 mice); (**C**). Ratio Firmicutes/Bacteroidetes. The ratio is represented by box and whiskers plots (median and quartile values), Kruskal–Wallis rank sum test (pairwise Wilcoxon rank sum post hoc test).

The composition of gut microbiota was assessed at week 12 of the food intervention. Permutational multivariate analysis of variance (PERMANOVA) analysis showed a minor insignificant effect of cage repartition (explained variation 9.9%, *p* = 0.112), and variation is explained by diet intervention (56%, *p* = 0.001) (Figure 5B). We observed decreased bacterial diversity and richness in the HFD group compared to both CD-fed (*p* ≤ 0.05) and *L. plantarum*-treated groups (Figure 5A).

The most abundant phyla were Firmicutes (65% ± 17%) and Bacteroidetes (28% ± 15%), followed by Proteobacteria (4% ± 2%) and Verrucomicrobia (2% ± 2%). As shown in Figure 5C, PBS-HFD mice harbored a higher ratio of Firmicutes to Bacteroidetes than the CD-fed group (*p* ≤ 0.01). Supplementation with this strain of *L. plantarum* alleviated this increase (*p* = 0.08).

**Figure 6:**
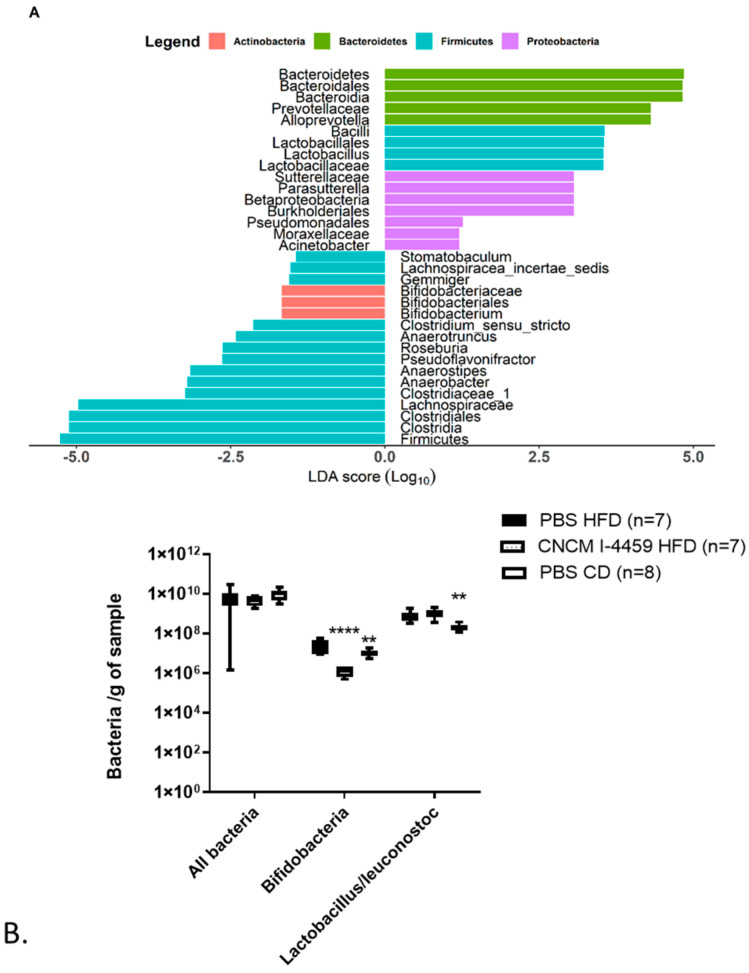
***L. plantarum* CNCM I −4459 modulated microbiota.** (**A**). Differential taxa abundance between PBS-HFD and *L. plantarum* CNCM I−4459-HFD. The LEfSe algorithm uses the effect size of each differentially abundant feature, and significance is subsequently investigated using a set of pairwise tests using the (Unpaired) Wilcoxon rank-sum test; (**B**). Measures of fecal bacteria by qPCR. Data represent box and whiskers plots (mean, minimal, and maximum values). Data were analyzed with Kruskal–Wallis test (Dunn’s post hoc test) and compared to PBS-administered HFD-fed mice. ** and **** represent, respectively, *p* < 0.01 and *p* < 0.0001.

At the genus level, the microbiota of HFD mice contained lower abundances of *Akkermansia*, *Barnesiella*, *Prevotella*, and *Stomatobaculum* and higher abundances of *Dorea*, *Moryella*, *Roseburia*, *Olsenella*, and *Blautia* (Supplementary Figure S7) compared to CD fed mice. Compared to PBS-HFD mice, treatment with *L. plantarum* induced slight modifications in the microbiota composition; specifically, 4 genera (*Alloprevotella*, *Lactobacillus, Parasutterella*, and *Acinetobacter*) were over-represented in mice treated with *L. plantarum* CNCM I −4459 (Figure 6A), and 10 genera, mainly belonging to phylum Firmicutes, were under-represented (*Stomatobaculum*, *Bifidobacterium*, *Anaerotruncus*, *Anaerobacter*, *Roseburia*, *Pseudoflavonifractor*, *Anaerostipes*, *Gemmiger*, *Lachnospiraceae incertae sedis*, and *Clostridium sensu stricto*).

For total bacterial load, Bifidobacteria and *Lactobacillus*/*Leuconostoc* groups were assessed by qPCR (Figure 6B). Our results showed an increase in *Lactobacillus/Leuconostoc* groups in HFD-fed mice (*p* ≤ 0.01) and an increase in Bifidobacteria in HFD-PBS mice (*p* ≤ 0.01). No modification was observed in the *Lactobacillus/Leuconostoc* group as a result of *L. plantarum* intervention; however, bacterial treatment decreased significantly in the Bifidobacteria group (*p* ≤ 0.0001).

### 3.5. L. plantarum CNCM I−4459 Did Not Affect Fecal End-Products of Fermentation

**Figure 7:**
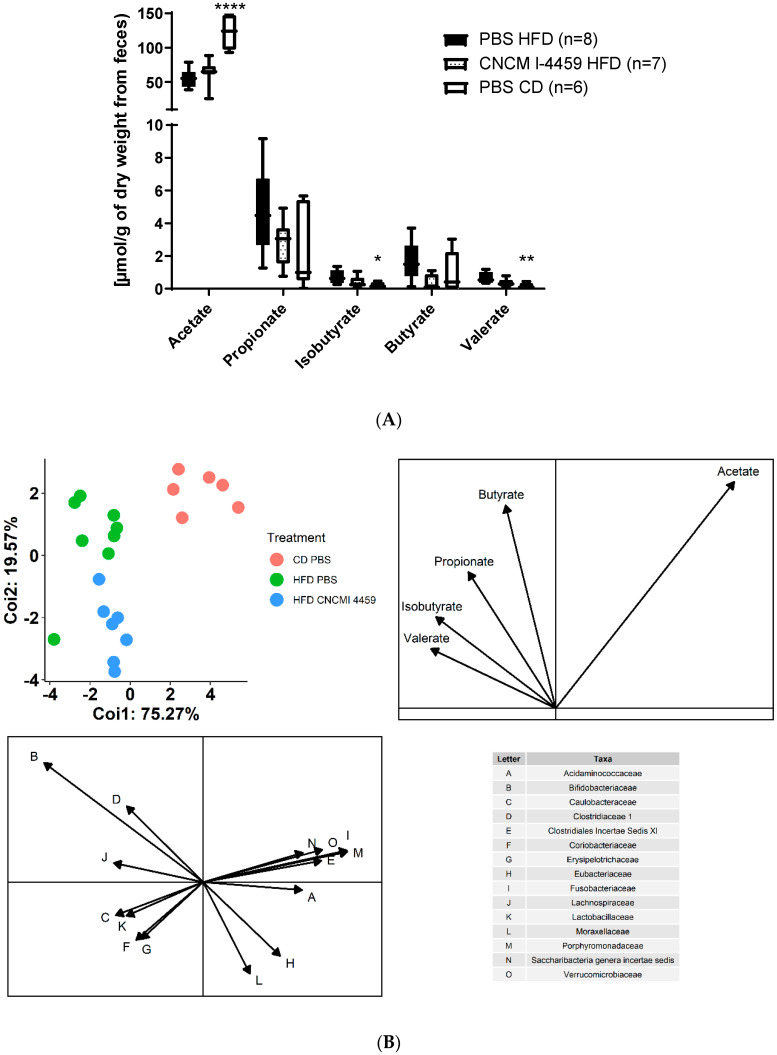
**Modulation of fecal short-chain fatty acids:** (**A**) Level of SCFA; data are represented as box and whiskers plots (mean, minimal, and maximum values). Data were analyzed with Kruskal–Wallis test (Dunn’s post hoc test) and compared to PBS-administered HFD-fed mice. *, **, and **** represent, respectively, *p* < 0.05, *p* < 0.01, and *p* < 0.0001; (**B**) Correlation between microbiota composition at family level and SCFA. Samples in red correspond to mice fed with CD, samples in blue correspond to mice fed with HFD and treated with *L. plantarum* CNCM I−4459, and samples in green correspond to mice fed with HFD.

From fecal samples, we measured concentrations of SCFAs (acetate, butyrate, valerate, and propionate) and the branched-chain fatty acid isobutyrate. Compared to the control group, HFD had significantly reduced concentrations of acetate (*p* ≤ 0.0001) and increased concentrations of isobutyrate (*p* ≤ 0.05) and valerate (*p* ≤ 0.01). *L. plantarum* CNCM I−4459 supplementation to the HFD diet tended to reduce concentrations of valerate even though it did not reach significance (Figure 7A). In light of the high positive correlation between fecal butyrate concentration and the abundance of the bacterial families Bifidobacteriaceae and Clostridiaceae (rho: 0.54 and 0.53; *p* ≤ 0.05; Figure 7B), it is possible that the observed decrease in butyrate could be explained by the reduced relative abundance of *Bifidobacterium*, releasing substrates for butyrate-producers such as *Clostridium*. However, when a co-inertia analysis combining both SCFAs and microbiota compositions was performed, samples did not cluster according to diet or treatment (Figure 7B).

### 3.6. Correlation between Microbiota Composition at Family-Level and Physiological Parameters

**Figure 8:**
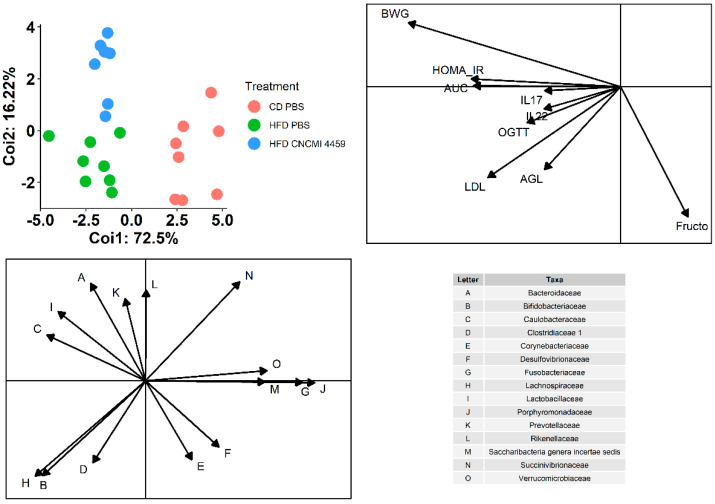
**Effect of HFD on the gut microbiota and metabolic parameters correlation.** Samples in red correspond to mice fed with CD, samples in blue correspond to mice fed with HFD and treated with *L. plantarum* CNCM I−4459, and samples in green correspond to mice fed with HFD.

In order to evaluate the correlation between metabolic parameters and microbial composition, PCAs were individually carried out on microbiota composition at (family level) and host metabolic composition and then subjected to co-inertia analysis in order to highlight associations between the two datasets. Interestingly, mice clustered according to probiotic treatment (16%), diet (73%), and bacterial composition (Figure 8). As expected, HFD correlated positively with all metabolic parameters (body weight gain, OGTT, LDL, and FFA levels), including inflammation IL-17 and IL-22 (Figure 8). At the family level, the abundance of Bacteroidaceae (rho: −0.37; *p*≥ 0.05) and Prevotellaceae (rho: −0.35; *p* ≥ 0.05) was negatively correlated with fructosamine levels. The abundance of Lachnospiraceae (rho: 0.73; *p* ≤ 0.05) and Bifidobacteriaceae (rho: 0.63; *p* ≤ 0.05) positively correlated with LDL-c levels, whereas Succinovibrionaceae (rho: 0.45; *p* ≤ 0.05) negatively correlated with LDL-c (Figure 8). Additionally, Clostridiaceae (rho: 0.30; *p* ≥ 0.05) positively correlated with FFA.

## 4. Discussion

In the present work, we aimed to assess the beneficial health effect of *L. plantarum* strain CNCM I−4459 on HFD-fed mice. Although we detected no effect of *L. plantarum* treatment on body weight or food intake, the probiotic treatment improved a few metabolic parameters.

HFD-fed mice are known to develop dyslipidemia with elevated serum lipid levels [38]. Circulating fatty acids enter directly into the liver for lipid synthesis [39]. Here, *L. plantarum* treatment stabilized LDL-c serum levels to concentrations that were similar to those of CD-fed mice. Usually, LAB decreases hyperlipidemia via activation of β-oxidation and/or the inhibition of lipogenesis in the liver [21,40]. However, Yoo et al. [41] demonstrated that a combination of strains of *Latilactobacillus curvatus* and *L. plantarum* decreased hepatic lipid droplets and resulted in weight loss through the downregulation of β-oxidation and fatty acid synthesis. Interestingly, here, the expression of lipogenic genes (*Fas* and *Plin*), lipolytic genes (*Cpt1-α*), and the insulin-dependent lipogenic inhibitor of *Srebp* (*Insig-2*) were downregulated in *L. plantarum* treated mice. Thus, this bacterial strain might lower hepatic lipid metabolism even if the liver dyslipidemia should be addressed in the future to confirm these observations. Additionally, obesity is characterized by an excess of fat stored in adipose tissue, and thus, the regulation of lipid metabolism in adipose tissue is a crucial target for research. Studies have revealed that a good prognostic indicator for obesity is higher adipocyte expression of oxidative genes [18,19]. Among these, Ppar-α (peroxisome proliferator-activated receptor alpha) is a key regulator of fatty acid oxidation and adipocyte differentiation [42]. Here, interestingly, mice fed the HFD and treated with *L. plantarum* showed increased *Ppar-α* expression in adipose tissue.

Obesity is associated with disturbances in glucose tolerance, leading to insulin resistance and type-2 diabetes [43]. The liver regulates glucose homeostasis and is the site of uncontrolled gluconeogenesis that can trigger hyperglycemia. In 1999, Rajas et al. [44] described the additional and crucial role played by *G6pase* (glucose 6 phosphatase, a limiting enzyme in gluconeogenesis) in the small intestine, particularly in diabetic rats. Notably, we observed here that treatment with *L. plantarum* inhibited glucose metabolism and lowered the expression of *G6pase* in the liver and the ileum. *G6pase* expression was not observed in colon samples, which was unsurprising since its expression is known to decrease along the gut [45]. Recently, Balakumar et al. described an improvement in glucose tolerance and insulin sensitivity in HFD-fed mice as a result of probiotic intervention. They also reported no modifications in glucose transport proteins, with the exception of *Glut-4* (whose expression is dependent on insulin) [43]. We hypothesize that in a diabetic state (as observed in HFD-fed mice), *G6pase* and *Gcg-1* expression are increased, which enhances plasmatic concentrations of glucose and insulin. Treatment with *L. plantarum* stabilized glucose levels by inhibiting glucose metabolism and transport in an insulin-dependent manner. Indeed, probiotic treatment significantly reduced glucose levels (including fasting levels) during oral glucose tolerance tests and consequently the AUC. These data suggest that *L. plantarum* prevented the development of hyperglycemia in HFD-fed mice.

HFD-fed mice are characterized by inflammation phenotype [4,46]. Indeed, several studies have reported increased levels of TNF-α, IL1-β, and IL-6 in adipose tissue that can lead to insulin resistance [47,48]. This inflammation does not originate only from the adipose tissues but can also spread to the intestine [47]. Although the present study confirmed that the HFD treatment induced systemic inflammation, a difference in TNF-α level was not found in the serum of *L. plantarum*-treated mice compared to PBS-HFD-fed mice or in intestinal sections. IL-17 is a pro-inflammatory cytokine that has been reported to be upregulated in obese humans [49,50] as well as in mice with diet-induced obesity [51]. Our data are consistent with several studies that have reported substantial regulation of inflammatory pathways in the small intestine [47,48] by administration of probiotics. *L. plantarum* CNCM I−4459 tends to mitigate the IL-17 ileal inflammation.

A strong connection between inflammation, epithelium integrity, and oxidative stress has been reported in the literature [49]. HFD-fed mice are characterized by impairments to permeability that are related to the downregulation of tight junction proteins (especially ZO-1). Here, oral administration of *L. plantarum* CNCM I−4459 significantly increased ZO-1 expression compared with PBS-HFD mice. In parallel, the expression of junction proteins in the ileum was reduced since no significant modulation was observed compared to PBS-CD mice. Interestingly, although HFD is known to disturb epithelial junctions, we found here that, overall, HFD intervention increased the expression of junction genes. The contrast between measurements of gene expression and protein levels suggests a compensatory effect that maintains protein levels and restores gut integrity. Here, these modifications in ileal junction proteins were correlated with a slightly lower level of IL-22. There are some discrepancies in the literature about the impact of metabolic syndromes on IL-22 secretion. Some data report that IL-22 modulates gut epithelium integrity and homeostasis, and recombinant IL-22 has been found to restore the barrier and metabolic parameters in HFD-fed mice [52,53]. In contrast, Garidou et al. [54] described a lower level of intestinal IL-22. This contrast might be explained in part by the different locations (adipocyte, splenocyte, or proximal vs. distal intestine) targeted by the different studies [53,54,55]. Taken together, the modifications observed as a result of treatment with *L. plantarum* CNCM I−4459 argue in favor of a restoration of the physiological state since this treatment seemed to normalize levels of both tight junction proteins and IL-22 to those observed in CD-fed mice. Additionally, these regulations seemed to be independent of oxidative stress since no modification was observed for Nrf-2. However, only Nrf-2 gene expression was assessed, and not oxidative activity; thus, these data should be interpreted with caution.

Finally, accumulating reports reinforce the relationship between microbiota dysbiosis and metabolic disorders, such as diet-related obesity [56,57]. Here, we demonstrate that treatment with *L. plantarum* CNCM I−4459 was able to partially reverse HFD-induced dysbiosis by increasing microbial richness. Interestingly, *L. plantarum* CNCM I−4459 counteracted the increase in the ratio of Firmicutes to Bacteroidetes that has been linked with obesity [1,2]. However, the probiotic intervention had no clear effect on microbial composition, as samples clustered only according to type of diet (as demonstrated via analyses of beta diversity). Notably, HFD induced the over-representation of Firmicutes, mainly *Oscillobacter, Dorea, Moryella, Lactobacillus, Roseburia*, and *Blautia*, and Actinobacteria, with *Olsenella*. Instead, genera such as *Akkermansia, Prevotella*, or *Barnesiella*, which have been associated with healthy phenotypes, were under-represented [58,59,60,61]. In most previous studies, HFD intervention induces a decrease in *Bifidobacterium* [46]. However, a few studies have reported, as we do here, an increase in this genus; this phenomenon has been explained mainly as a function of the bifidogenic activity of maltodextrin, which is present in higher amounts in HFD [40,62,63]. Treatment with *L. plantarum* CNCM I−4459 modulated microbial composition only slightly, with an increase in *Lactobacillus* (probably due to the daily oral administration of *L. plantarum* CNCM I−4459) and *Prevotella* and a decrease in *Roseburia, Anaerostipes*, and *Stomatobaculum* (Lachnospiraceae); *Anaerobacter* and *Anaerotruncus* (Clostridiaceae)*;* and *Bifidobacterium*.

Administration of HFD is commonly linked with alterations in SCFA content, as HFDs are low in fiber (one of the substrates for intestinal bacterial fermentation), and animals fed HFD exhibited modified levels of SCFAs [64]. In our model, HFD did not affect the major SCFA products (butyrate and propionate) but led to a decrease in acetate, along with significant increases in valerate and isobutyrate. Treatment with *L. plantarum* CNCM I−4459 tended to reduce fecal butyrate content, probably linked to lower butyrate producers such as *Roseburia* spp. Several studies have reported beneficial health effects of SCFAs on host physiology, especially butyrate (reviewed in [65]). However, it is not clear if SCFA modulation directly contributes to the obesity phenotype or if it is instead a consequence of microbial disturbance. A previous correlation analysis revealed a positive link between butyrate and members of the Bifidobacteriaceae and butyrate-producing Clostridiaceae [66], which here were both less abundant in mice treated with this strain of *L. plantarum*. In addition, and independently of a direct microbial effect on SCFA release, the increase in permeability observed in obese animals could favor intestinal absorption and consequently impact the quantification in fecal samples. Altogether, these data suggest that i) HFD significantly disturbed the composition of gut microbiota and ii) intervention with the probiotic strain restored these altered communities to their “healthy” composition.

All of the metabolic parameters measured here were positively correlated with the bacterial taxa that were enriched in HFD-fed mice. The bacterial families that were positively correlated with probiotic treatment (Lachnospiraceae, Bacteroidaceae, and Prevotellaceae) were negatively correlated to fructosamine levels. This is in agreement with previous work showing the beneficial effect of *Prevotella* on glucose tolerance [60]. In our study, FFA content was positively correlated with the abundance of Clostridiaceae, while LDL-c was positively correlated with Bifidobacteriaceae and Lachnospiraceae and negatively correlated with Succinivibrioceae. However, these data should be treated carefully because we performed our microbiota analysis on fecal samples, and therefore, we cannot account for any changes in composition that may have occurred in different regions of the gut. In the near future, it will be particularly interesting to determine if the microbial community is different in the ileal mucosa, where we observed several important physiological changes. Additionally, gut microbiota was only assessed at the taxonomic composition, and we cannot exclude that changes at the functional level (transcriptomic analysis) could explain the observed improvements. Of note, previous studies have also shown the effects of probiotic consumption on different hematological parameters in various animal models, such as mice, cats, dogs, etc. [67,68].

In conclusion, treatment with *L. plantarum* CNCM I−4459 improved global metabolic parameters that were compromised by HFD administration. Here, we show that the CNCM I−4459 probiotic strain can synergistically and moderately act at different levels to improve host metabolism and favor the maintenance of homeostasis. *L. plantarum* CNCM I−4459 tended to restore the microbial composition and resulting SCFAs (even no significant) content to healthy profiles (here reflected by CD-fed mice) rather than favoring the expansion of certain detrimental species, with the overall result being an alleviation of HFD-induced microbiota dysbiosis. Overall, *L. plantarum* CNCM I−4459 ameliorated the HFD-induced disturbance, with global effects on host metabolism and, in particular, a major impact on glucose sensitivity. However, our data are to consider a sex-dependent effect since our experiments were driven on male mice, and it was recently described that HFD intervention was mainly impacted in male subjects. Together, these observations elegantly illustrate the concept of “symbiostasis” (based on host homeostasis and host and microbiota symbiosis) and open new perspectives to leverage combined nutrition–host– microbiome strategies for the treatment and prevention of metabolic diseases, although additional experiments will be required to investigate further human applications.

## Figures and Tables

**Table 1:** **Effect of *L. plantarum* CNCM I−4459 on circulating lipids.** Data are represented as mean for 8 mice per group ± SEM. Data were compared to PBS-administered HFD-fed mice. *, **, **** represent a *p* < 0.05, *p* < 0.01, and *p* < 0.0001. TG, LDL-C, FFA, and TC were analyzed using one-way ANOVA, followed by Tukey’s multiple comparison.

Diet	HFD–PBS	CD–PBS	HFD—*L. plantarum* CNCM I−4459
TG (mmol/L)	1.21 ± 0.24	0.94 ± 0.15	1.17 ± 0.28
LDL-c (mmol/L)	0.42 ± 0.06	0.24 ± 0.04 ****	0.26 ± 0.04 ****
HDL-c (mmol/L)	2.67 ± 0.18	2.15 ± 0.17 *	2.76 ± 0.55
FFA (mmol/L)	1.3 ± 0.12	1.01 ± 0.38	1.09 ± 0.21
TC (mmol/L)	4.85 ± 0.43	3.64 ± 0.25 **	4.77 ± 0.94

## Data Availability

16S rRNA gene sequencing data are available under the NCBI BioProject accession number PRJNA663256.

## Supplementary Materials

The following supporting information can be downloaded at: https://www.mdpi.com/article/10.3390/bioengineering10101151/s1, Figure S1: Pyy mRNA expression in HUTU 80 cells. Bacteria were grown in MRS (Man, Rogosa and Sharpe medium, Oxoid CM0359) at 37 °C under pseudoaerobic conditions. Bacterial cultures (stationary phase) were centrifuged at 5000× *g* for 10 min. Bacterial pellets were washed twice with PBS and resuspended in Dulbecco’s Modified Eagle’s Medium (DMEM, Lonza, Basel, Switzerland) supplemented with a 100× concentration penicillin/streptomycin (PS, Gibco, Thermo Scientific, Illkirch-Graffenstaden, France) solution. Cells were stimulated with bacteria at MOI 40 (corresponding to 1.6 × 10^9^ CFU/mL) during 24 h. Total RNA was extracted with Qiashredders column and purified with RNeasy minikit (Qiagen, Courtaboeuf, France) following the manufacturer’s recommendations. RNA concentration was measured with a Nano-Drop spectrophotometer (NanoDrop Technologies, Wilmington, DE, USA). Briefly, cDNA synthesis was performed from 1 μg of RNA using the High Capacity cDNA Reverse Transcription Kit (Applied Biosystems, USA) according to the manufacturer’s instructions. The cDNAs were diluted to 20 ng/mL. RT-qPCRs were performed with Taqman probes (Life Technologies, France) according to the manufacturer’s instructions using an ABI Prism 7700 thermal cycler (Applied biosystems, USA) in a reaction volume of 25 μL. For each sample and each gene, PCR was performed in triplicate. To quantify and normalize the expression data, the ΔΔΔCt method was used using the geometric mean Ct value of Gapdh as reference endogenous genes. Figure S2: Diet composition. Figure S3: Assessment of food intake and satiety. A. Food intake. Mice were fed either with HFD (High-Fat Diet) or CD (Control Diet) and were orally administered with either PBS (control vehicle) or *L. plantarum* CNCM I −4459 (1 × 10^9^ CFU/day) for 12 weeks. Data represent mean ± SEM on two independent experiments; mRNA expression of anorexigenic genes (B) *Gcg1* in ileum; (C) *Gcg1* in colon; (D) *Pyy* in ileum and (E) *Pyy* in colon. Data are represented as Box and whiskers plots (mean, minimal and maximum values) and compared to PBS administered HFD fed mice. Data were analyzed with Kruskal−Wallis Test (Dunn’s post hoc test). * and ** respectively represent a *p* < 0.05 and *p* < 0.01. ns = no significant. Figure S4: Lipids metabolism in liver and adipose tissue. A. Hepatic mRNA *Cd36* expression; B. Hepatic mRNA Ppar*α* expression; C. Hepatic mRNA Chrebp expression; D. Hepatic mRNA Cyp7a1 expression; E. Adipocyte mRNA *Fas* expression; F. Adipocyte mRNA *Cpt1a* expression; G. Adipocyte mRNA *Ucp1-*α expression. Genes were measured in AT. Data are represented as Box and whiskers plots (mean, minimal and maximum values). Data were analyzed with Kruskal−Wallis Test (Dunn’s post hoc test) except for A (one-way ANOVA, followed by Tukey’s multiple comparison) and compared to PBS administered HFD fed mice for 8 mice per group. * represents a *p* < 0.05. ns = no significant. Figure S5: Assessment of inflammation in colon and jejunum. A. mRNA expression of *Tnf-α* in colon; B. TNF-α protein level in colon; C. IL-17 protein level in colon; D. mRNA expression of *Tnf-*α in jejunum; E. TNF-α protein level in jejunum; F. IL-17 protein level in jejunum. Data are represented as Box and whiskers plots (mean, minimal and maximum values). Data were analyzed with Kruskal−Wallis Test (Dunn’s post hoc test) except for D (one-way ANOVA) and compared to PBS administered HFD fed mice. Figure S6: Assessment of epithelial barrier homeostasis in different intestinal sections. A. mRNA expression of *Zo-1* (*Zonula Occludens-1*) in colon, B. Level of ZO-1 expression in colon, lines 1, 2 and 3 correspond respectively to a mice treated with *L. plantarum* CNCM I−4459 in HFD, PBS-HFD mice and PBS-CD mice, C. mRNA expression of *Claudin 5* in colon, D. mRNA expression of *Claudin 2* in colon, E. mRNA expression of *Occludin* in colon, F. mRNA expression of *Claudin 5* in ileum, G. mRNA expression of *Occludin* in ileum, H. mRNA expression of *Zo-1* in ileum, I. mRNA expression of *Claudin 2* in ileum, J. Ileal IL-22 level and K. mRNA expression of *Nrf2* in ileum. Data are represented as Box and whiskers plots (mean, minimal and maximum values). Data were analyzed with Kruskal−Wallis Test (Dunn’s post hoc test) except for C, D, E, I and K (one-way ANOVA, followed by Tukey’s multiple comparison) and compared to PBS administered HFD fed mice for 8 mice per group. * and ** respectively represent a *p* < 0.05 and *p* < 0.01. ns = no significant. Figure S7: Modulation of fecal microbiota in HFD-fed mice compared to CD−fed mice. The LEfSe algorithm uses the effect size of each differentially abundant feature and significance is subsequently investigated using a set of pairwise tests using the (Unpaired) Wilcoxon rank-sum test.
