# Supplementary material for: Impact of Oral Administration of Lactiplantibacillus plantarum Strain CNCM I−4459 on Obesity Induced by High-Fat Diet in Mice
Source: Bioengineering (Basel). 2023 Oct 1;10(10):1151. doi: 10.3390/bioengineering10101151 (PMC10604482; doi:10.3390/bioengineering10101151)
Supplement: Supplementary file 1 [file bioengineering-10-01151-s001.zip › bioengineering-2531001-supplementary.pdf]

Impact of oral administration of *Lactiplantibacillus plantarum* strain CNCM I-4459 on obesity induced by high-fat diet in mice

SUPPLEMENTARY DATA

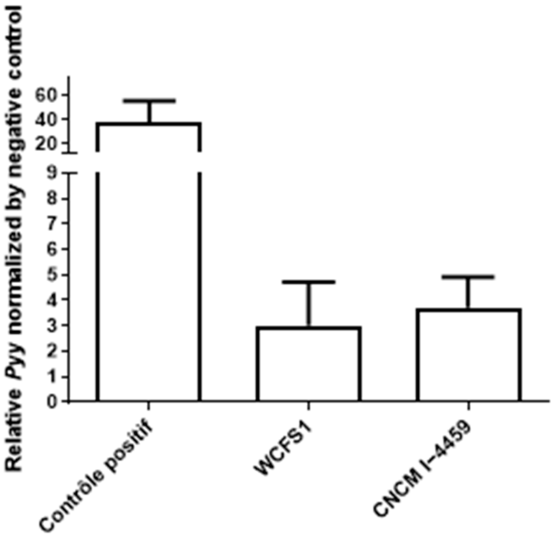

Figure S1. Pyy mRNA expression in HUTU 80 cells. Bacteria were grown in MRS (Man, Rogosa and Sharpe medium, Oxoid CM0359) at 37°C under pseudoaerobic conditions. Bacterial cultures (stationary phase) were centrifuged at 5,000x g for 10 minutes. Bacterial pellets were washed twice with PBS and resuspended in Dulbecco’s Modified Eagle’s Medium (DMEM, Lonza, Basel, Switzerland) supplemented with a 100 × concentration penicillin/streptomycin (PS, Gibco, Thermo Scientific, Illkirch-Graffenstaden, France) solution. Cells were stimulated with bacteria at MOI 40 (corresponding to 1.6 x 10^9 CFU/ml) during 24h. Total RNA was extracted with Qiashredders column and purified with RNeasy minikit (Qiagen, Courtaboeuf, France) following the manufacturer's recommendations. RNA concentration was measured with a Nano-Drop spectrophotometer (NanoDrop Technologies, Wilmington, USA). Briefly, cDNA synthesis was performed from 1µg of RNA using the High Capacity cDNA Reverse Transcription Kit (Applied Biosystems, USA) according to the manufacturer's instructions. The cDNAs were diluted to 20ng/ml. RT-qPCRs were performed with Taqman probes (Life technologies, France) according to the manufacturer's instructions using an ABI Prism 7700 thermal cycler (Applied biosystems, USA) in a reaction volume of 25µl. For each sample and each gene, PCR was performed in triplicate. To quantify and normalize the expression data, the ΔΔΔCt method was used using the geometric mean Ct value of Gapdh as reference endogenous genes.

HFD

Gross Energy (GE) 25.0 MJ/kg

Metabolizable Energy (ME) [1)] 21.6 MJ/kg

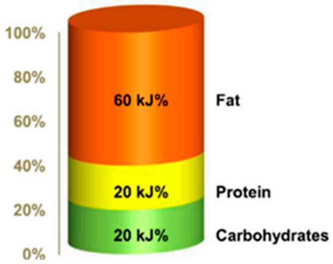

Crude Nutrients	[%]
Crude protein (N x 6.25)	24.4
Crude fat	34.6
Crude fibre	6.0
NDF	5.2
ADF	1.9
Crude ash	5.4
Starch	0.1
Sugar	9.4
N free extracts	26.3

Feed composition	%
Casein	27.700
Corn starch	——
Maltodextrin	15.600
Sucrose	8.490
Cellulose powder	6.000
L-Cystine	0.350
Vitamin premix	1.000
Mineral & trace element premix	6.000
Choline chloride (50 % choline)	0.250
Butylated hydroxytoluene	0.010
Beef tallow, premier Jus	31.500
Soybean oil	3.100

CD

Gross Energy (GE) 18.1 MJ/kg

Metabolizable Energy (ME) [1)] 15.1 MJ/kg

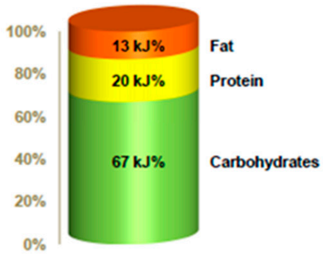

Crude Nutrients	[%]
Crude protein (N x 6.25)	18.2
Crude fat	5.1
Crude fibre	8.0
Crude ash	5.3
Starch	37.3
Sugar	**11.0**
N free extracts	60.1

Feed composition	%
Casein	20.700
Corn starch	38.850
Maltodextrin	10.000
Sucrose	10.000
Cellulose powder	5.000
Lignocellulose powder	3.000
L-Cystine	0.250
Vitamin premix	1.000
Mineral & trace element premix	6.000
Choline chloride (50 % choline)	0.200
Soybean oil	5.000

Figure S2. Diet composition.

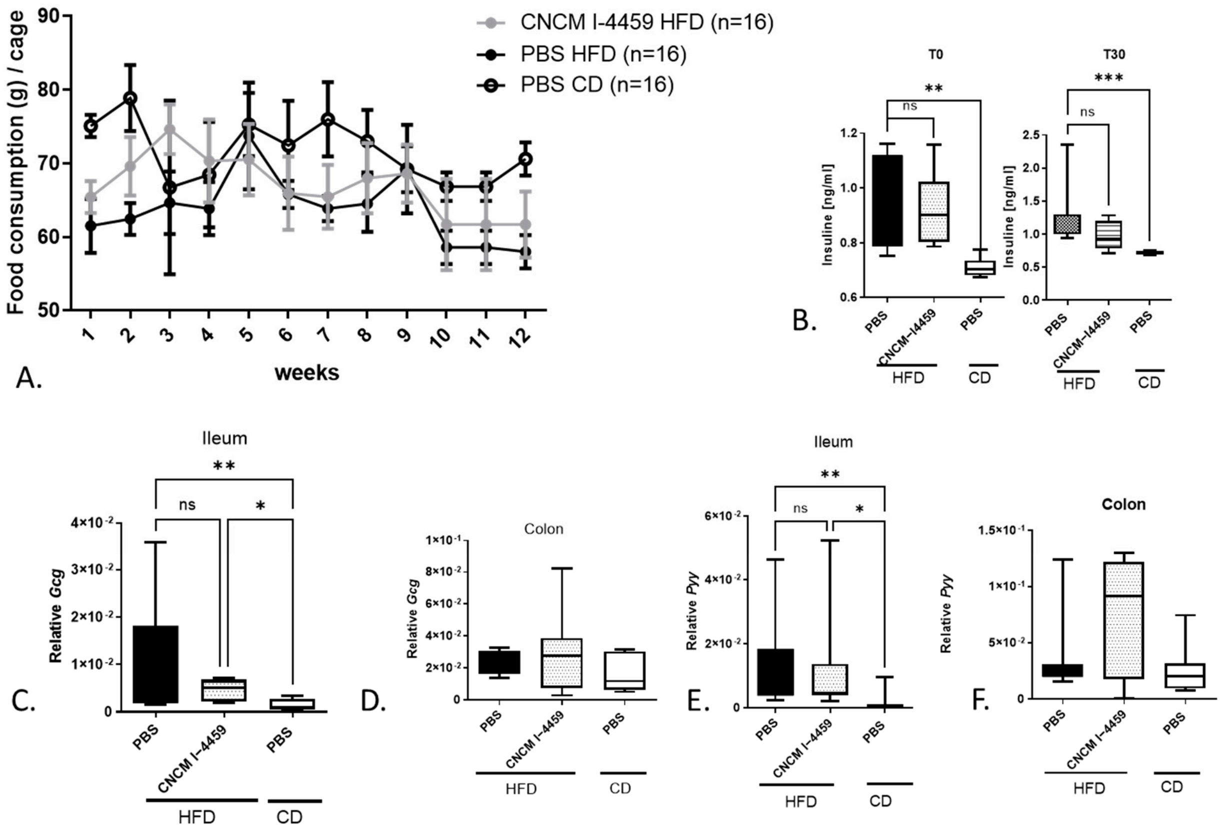

Figure S3: **Assessment of food intake and satiety**. A. Food intake. Mice were fed either with HFD (High-Fat Diet) or CD (Control Diet) and were orally administered with either PBS (control vehicle) or *L. plantarum* CNCM I −4459 (1×10^9 CFU/day) for 12 weeks. Data represent mean ± SEM on two independent experiments; mRNA expression of anorexigenic genes (B) *Gcg1* in ileum; (C) *Gcg1* in colon; (D) *Pyy* in ileum and (E) *Pyy* in colon. Data are represented as Box and whiskers plots (mean, minimal and maximum values) and compared to PBS administered HFD fed mice. Data were analyzed with Kruskal − Wallis Test (Dunn's post hoc test). * and ** respectively represent a $p < 0.05$ and $p < 0.01$. ns= no significant.

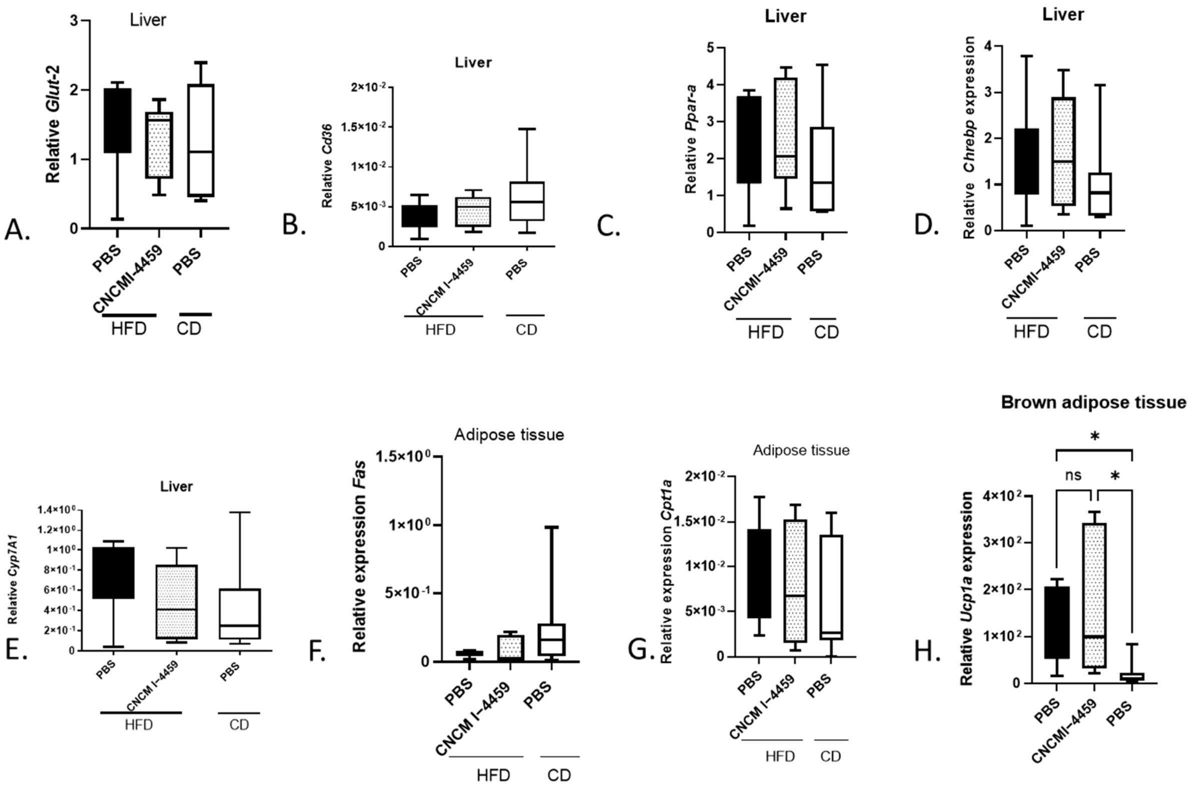

Figure S4: Lipids metabolism in liver and adipose tissue. A. Hepatic mRNA *Cd36* expression; B. Hepatic mRNA Ppar*α* expression; C. Hepatic mRNA Chrebp expression; D. Hepatic mRNA Cyp7a1 expression; E. Adipocyte mRNA *Fas* expression; F. Adipocyte mRNA *Cpt1a* expression; G. Adipocyte mRNA *Ucp1*−α expression. Genes were measured in AT. Data are represented as Box and whiskers plots (mean, minimal and maximum values). Data were analyzed with Kruskal−Wallis Test (Dunn's post hoc test) except for A (one-way ANOVA, followed by Tukey's multiple comparison) and compared to PBS administered HFD fed mice for 8 mice per group. * represents a $p < 0.05$. ns= no significant.

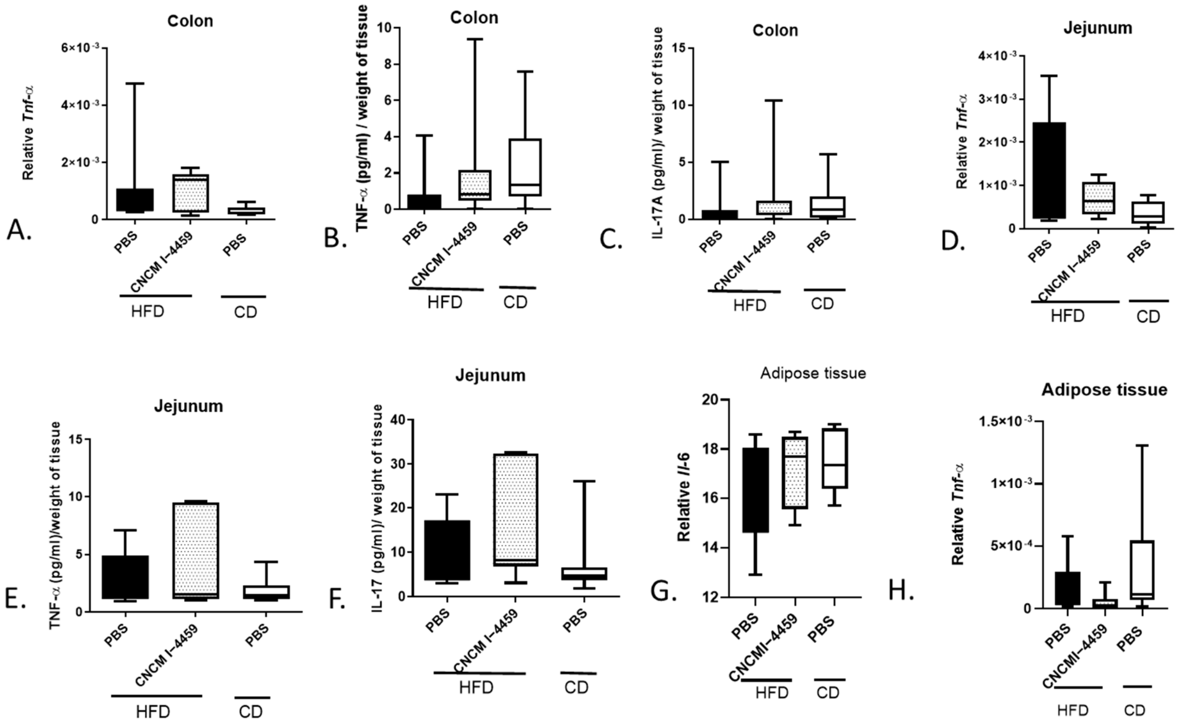

Figure S5: Assessment of inflammation in colon and jejunum. A. mRNA expression of *Tnf−α* in colon; B. TNF−α protein level in colon; C. IL−17 protein level in colon; D. mRNA expression of *Tnf*−α in jejunum; E. TNF−α protein level in jejunum; F. IL−17 protein level in jejunum. Data are represented as Box and whiskers plots (mean, minimal and maximum values). Data were analyzed with Kruskal−Wallis Test (Dunn's post hoc test) except for D (one-way ANOVA) and compared to PBS administered HFD fed mice.

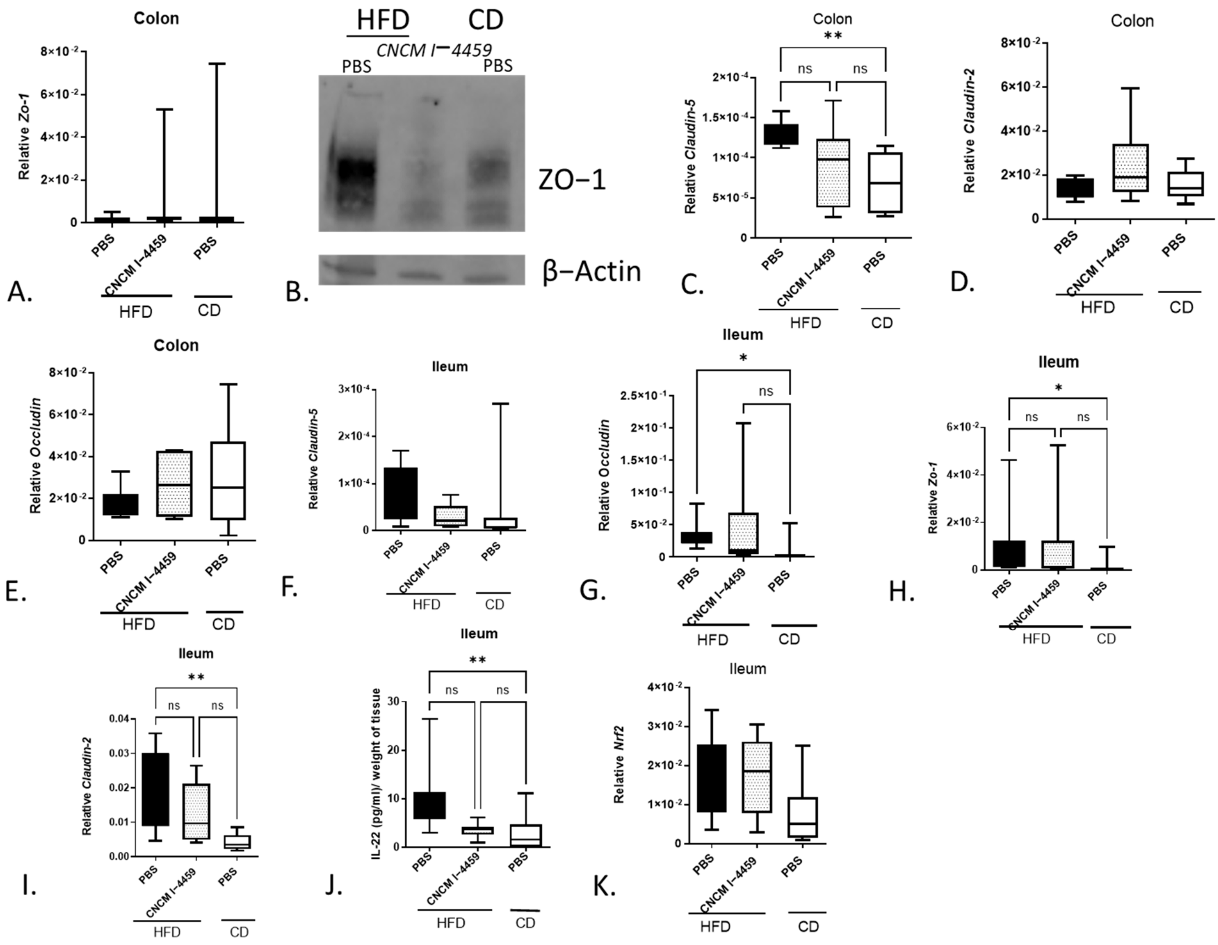

Figure S6: Assessment of epithelial barrier homeostasis in different intestinal sections. A. mRNA expression of *Zo−1* (*Zonula Occludens − 1*) in colon, B. Level of ZO−1 expression in colon, lines 1, 2 and 3 correspond respectively to a mice treated with *L. plantarum* CNCM I−4459 in HFD, PBS-HFD mice and PBS-CD mice, C. mRNA expression of *Claudin 5* in colon, D. mRNA expression of *Claudin 2* in colon, E. mRNA expression of *Occludin* in colon, F. mRNA expression of *Claudin 5* in ileum, G. mRNA expression of *Occludin* in ileum, H. mRNA expression of *Zo −1* in ileum, I. mRNA expression of *Claudin 2* in ileum, J. Ileal IL-22 level and K. mRNA expression of *Nrf2* in ileum. Data are represented as Box and whiskers plots (mean, minimal and maximum values). Data were analyzed with Kruskal−Wallis Test (Dunn's post hoc test) except for C, D, E, I and K (one-way ANOVA, followed by Tukey's multiple comparison) and compared to PBS administered HFD fed mice for 8 mice per group. * and ** respectively represent a $p < 0.05$ and $p < 0.01$. ns= no significant.

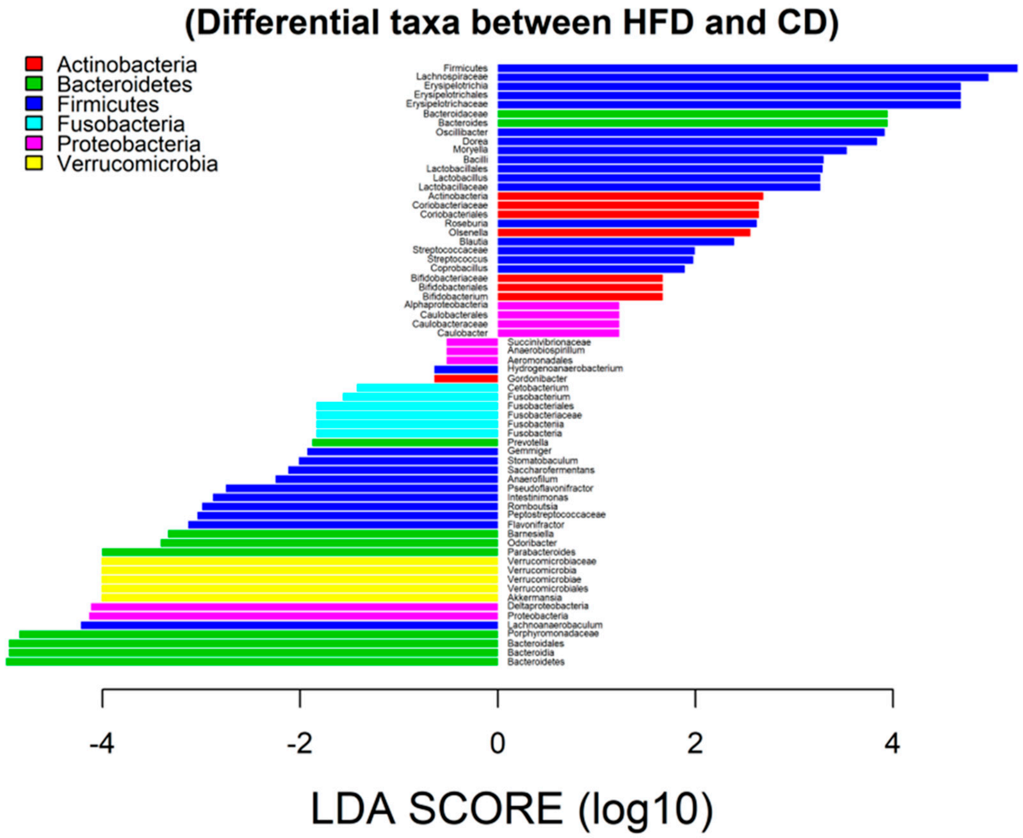

Figure S7: Modulation of fecal microbiota in HFD-fed mice compared to CD−fed mice. The LEfSe algorithm uses the effect size of each differentially abundant feature and significance is subsequently investigated using a set of pairwise tests using the (Unpaired) Wilcoxon rank-sum test.
